# Forward Flux Sampling of Polymer Desorption Paths from a Solid Surface into Dilute Solution

**DOI:** 10.3390/polym12102275

**Published:** 2020-10-03

**Authors:** Kyle J. Huston, Christina E. Rice, Ronald G. Larson

**Affiliations:** 1 Department of Chemical Engineering, University of Michigan, Ann Arbor, MI 48109-2136, USA; khuston@gmail.com (K.J.H.); cerice@umich.edu (C.E.R.); 2 Department of Mechanical Engineering, University of Michigan, Ann Arbor, MI 48109-2136, USA

**Keywords:** polymer desorption, forward flux sampling, Langevin dynamics simulations

## Abstract

We compute desorption rates for isolated polymers adsorbed to a solid wall with a rare event sampling technique called multilevel splitting, also known as forward flux sampling. We interpret computed rates with theories based on the conjecture that the product $\frac{t_{\mathrm{des}}D}{R_{g}^{2}}$ of the desorption time $t_{\mathrm{des}}$ and diffusivity D divided by squared radius of gyration $R_{g}$ scales with exp(*h/R_g_*) where *h* is the equilibrium ratio of adsorbed surface concentration of polymer Γ to bulk concentration of polymer c. As the polymer–wall interaction energy is increased, the slope of $ln\left(\frac{t_{\mathrm{des}}D}{R_{g}^{2}}\right)$ vs. $\frac{NV_{MF}}{k_{B}T}$ nearly approaches unity, as expected for strongly-adsorbing chains, where *N* is the degree of polymerization and $V_{MF}$ is the height-averaged monomer–wall interaction energy for a strongly adsorbed chain. However, we also find that this scaling law is only accurate when adsorption strength per monomer exceeds a threshold value on the order of 0.3–0.5 k_B_T for a freely jointed chain without or with excluded volume effects. Below the critical value, we observe that $\frac{t_{\mathrm{des}}D}{R_{g}^{2}}$ becomes nearly constant with *N*, so that $t_{\mathrm{des}}\propto N^{\alpha }$, with α≈2. This suggests a crossover from “strong” detachment-controlled to a “weak” diffusion-controlled desorption rate as *V*_MF_/k_B_T drops below some threshold. These results may partially explain experimental data, that in some cases show “strong” exponential dependence of desorption time on chain length, while in others a “weak” power-law dependence is found. However, in the “strong” adsorption case, our results suggest much longer desorption times than those measured, while the reverse is true in the weak adsorption limit. We discuss possible reasons for these discrepancies.

## 1. Introduction

To the best of our knowledge, the rate of desorption of an isolated chain from an interface has not been predicted using detailed theory or simulation, and even experiments measuring desorption rates are rare. [1,2,3]. In their textbook, Fleer et al. noted that in most cases, even in the absence of a kinetic barrier to desorption, polymer adsorbed at levels up to a significant fraction of saturation of the surface yields an equilibrium concentration in solution too small to drive sufficiently fast diffusion to be measurable [4]. The reason for the minuscule rate of desorption of long polymers from surfaces is that, if each monomer can adsorb with an adsorption free energy $\varepsilon_{PW}$ that is an appreciable fraction of $k_{B}T$ or more, then the total adsorption energy of the polymer with N monomers will be of order $N\varepsilon_{PW}$ >> $k_{B}T$. The chain will then readily pay the entropic penalty to adsorb enough monomers that desorption of a chain becomes an exceedingly rare event. There are, however, situations in which one should expect exceptions. First, the adsorption energy per monomer may be very feeble, $\varepsilon_{PW}$ << $k_{B}T$, in which case even chains of modestly high molar mass are bound with energies $N\varepsilon_{PW}$ < 10 $k_{B}T$, or so. These chains could desorb at a measurable rate. A second possibility is that only a fraction of the monomers can adsorb. This could occur for a variety of reasons. The polymer could be a heteropolymer, such as a protein in which only very hydrophobic, or hydrophilic, residues can bind. The adsorption might be due to charge interactions, wherein only charged monomers are attracted to charged sites on the surface, and if charge density is low on both polymer and surface, adsorption might be relatively weak. A case where this is known to be relevant is that of polyelectrolytes that adsorb onto either a bare surface or one coated with polymer, such as occurs in the layer-by-layer (LbL) process. Depending on pH and salt concentration, there may be a relatively small number of charges per polymer able to bind to the pre-existing layer, thus making the adsorption partially reversible, as in fact is experimentally well known in LbL coating [5]. A related situation might be one in which monomers stick to a surface by van der Waals interactions, but are electrostatically repelled from the surface. In this case, the total free energy of adsorption is reduced, allowing faster desorption. Yet another situation might be one in which desorption somehow becomes cooperative, in that monomers that desorb are somehow kept from re-adsorbing, allowing monomers to desorb sequentially. This situation might arise if there is some force, for example, a hydrodynamic force, that acts on the polymers, so that desorbed monomers have little chance to re-adsorb, and so are removed sequentially from the surface rather than needing many monomers to simultaneously overcome their desorption barriers [6].

Thus, while the simplest case of desorption of long chains of strongly-adsorbing monomers is well known to have trivial, negligible, desorption kinetics on experimental time scales, there are interesting situations in which we do not expect this to hold, and a method to calculate the actual rate may be important. Even in cases for which desorption is likely to be exceedingly slow, there may be reasons to nonetheless wish to know the rate. For example, polymers coating implanted materials in the body may be dangerous or toxic, and one wishes to be confident that their desorption rate is completely negligible. Since calculations of desorption rates are, with the few exceptions described below, almost unknown in the literature, we wish to advance understanding of desorption by applying the rare-event sampling method forward flux sampling (FFS) to compute desorption rates for uncharged homopolymers in the absence of flow or any external field. We test chains of varying length, varying polymer–wall interaction energy, and various internal potentials governing chain configuration. A rare-event sampling method, such as FFS, is needed to determine the desorption rate, since direct simulation will almost never reveal even a single desorption event for strongly bound chains, even when using coarse-grained simulation methods.

In the remainder of this Introduction, we position our work within the existing literature. First, we review experimental studies of polymer desorption from solid/liquid interfaces. Second, we review theories of polymer adsorption thermodynamics and desorption rates. Third, we review previous molecular simulations of polymer desorption. Then, we describe the forward flux sampling method, how its accuracy is analyzed, and modeling details. Then, we move onto the Results, Discussion, and Conclusions.

### 1.1. Experimental Measurements of Desorption Rate for Adsorbed Chains

While there are many experimental studies of polymer adsorption onto solid–liquid interfaces, including measurements of adsorption isotherms and of rates of adsorption, few experimental studies provide quantitative measurements of desorption rates. Studies by Granick and coworkers [2,3] from the early 1990s used Fourier-transform infrared spectroscopy in total internal reflection mode to measure desorption rates in two systems. The first, by Frantz and Granick [2] studied the desorption of protio-polystyrene from silicon surfaces into the cyclohexane solvent, driven by replacement by the isotopic deutero-polystyrene. Desorption times ranged from minutes to 100 h, exponentially increasing with increasing chain length from *N* = 1000 to 12,000 monomers [2]. In the second study, by Johnson, Douglas, and Granick [3], the adsorption of polystyrene (PS) from into carbon tetrachloride solvent onto an oxidized silicon surface was followed immediately by adsorption of stronger-binding poly(methyl methacrylate) (PMMA), which formed an “overlayer” that gradually displaced the already adsorbed polystyrene, leading to its desorption [3]. In this second study, a power-law scaling of desorption time with degree of polymerization *N* with exponent around 2.3 was observed; this was attributed to relatively rapid displacement of PS by stronger-binding PMMA, so that the time for “desorption” was dominated by diffusion of the desorbed chain through an “overlayer” of entangled polymers. However, the times required for desorption ranged from a minute or so up to 15 h as chain lengths increased from ~100 to ~1000 monomers. These times are much too long to be simple diffusion times, even diffusion through an entangled mesh, given the solvated state of the polymer and the short distances involved (<1 μm). In both of these studies, adsorbed chains were not dilute on the surface, and competition for adsorption sites likely accelerated the desorption rate.

In the only experimental study we know of where chains were dilute and isolated from each other, that of Skaug, Mabry, and Schwartz [1], desorption was observed by optical imaging of stained poly(ethylene oxide) chains. The chains were observed “hopping” from one fixed location to another along the surface, with a hopping time proportional to *N*^0.6^ with times ranging from 0.1 to 1 s, with *N* ranging from 40 to 1000.

### 1.2. Theories of Isolated Polymer Adsorption Thermodynamics and Expected Desorption Rate for Strongly Adsorbed Chains 

In the limit of strong adsorption, an isolated polymer chain flattens onto the surface like a pancake into a layer of thickness comparable to that of a Kuhn length of the polymer chain, and each monomer experiences the same “mean-field,” or height-averaged, potential *V*_MF_, which in this case is the thermodynamic (i.e., Boltzmann-weighted) average interaction between the wall and each monomer, i.e.,

(1) $$V_{MF}\left(\sigma_{PW},\epsilon_{PW}\right)=\frac{1}{Z}\int_{z=0}^{2\sigma_{PW}}V_{W}\left(z;\sigma_{PW},\varepsilon_{PW}\right)\exp\left(-\frac{1}{k_{B}T}V_{W}\left(z;\sigma_{PW},\varepsilon_{PW}\right)\right)\;dz$$

where $V_{W}$ is a potential that depends on the distance z between the monomer and the wall and $\sigma_{PW}$ and $\varepsilon_{PW}$ are parameters of the Lennard Jones-like monomer-wall potential described below. In the above, *Z* is the normalization constant, which is the same integral without $V_{W}\left(z;\sigma_{PW},\varepsilon_{PW}\right)$. Note that $V_{W}\left(z;\sigma_{PW},\varepsilon_{PW}\right)$ is close to zero at $z=2\sigma_{PW}$. For convenience in plotting results, we have taken here the attractive potential $V_{MF}$ to be positive by introducing the minus sign before the integral. Our underlying kinetic scaling assumption is that the logarithm of the average desorption time $t_{\mathrm{des}}$, normalized by the diffusivity D and radius of gyration $R_{g}$ of the free chain, is related to the free energy *F* of the adsorbed state relative the free state, via:

(2) $$\ln\left(\frac{t_{\mathrm{des}}D}{R_{g}^{2}}\right)\propto -\frac{F}{k_{B}T}$$

Ignoring entropic contributions to the adsorption free energy F of the adsorbed pancake, we obtain a simple “strong-adsorption” scaling law from the number of monomers N confined in the mean-field potential *V*_MF_:

(3) $$\ln\left(\frac{t_{\mathrm{des}}D}{R_{g}^{2}}\right)\propto -\frac{NV_{MF}\left(z;\sigma ,\varepsilon \right)}{k_{B}T}$$

Eisenriegler, Kremer, and Binder (1982) derived scaling exponents for the desorption time from the Gaussian polymer field theory for an ideal chain near the crossover point from adsorption to depletion by renormalization group theory [7]. For a self-avoiding walk, at the crossover point, the number of adsorbed monomers scales as $N_{\mathrm{train}}\propto N^{\phi }$, and the exponent ϕ relating the number of adsorbed monomers to the total number of monomers *N* in the chain is approximately 0.6, as one might expect at the cross-over point where the chain configuration and monomer density are relatively unaffected by the presence of the wall. Thus, at the cross-over point, the fraction of monomers that are adsorbed shrinks to zero as the chain becomes longer. These studies may be relevant to the weakly adsorbed limit.

### 1.3. Previous Molecular Simulations of Polymer Desorption

Published works that are the most similar to what we propose here include that of Wang, Rajagopalan, and Mattice [8], who performed Monte Carlo (MC) simulations on a cubic lattice of the desorption of an adsorbed film of multiple polymers, from a surface. Their results show a desorption time scaling as *N*^2.5^, suggestive of diffusive control of desorption, with desorption time ranging from 400 to 400,000 MC steps for chain lengths ranging from 5 to 85 units. While consistent with one of the experimental studies of Johnson et al. [3], the chains in the simulations of Wang et al. were too short to be entangled, and hence their results, while showing a similar scaling law as in [3], must be explained by simple diffusion, without entanglement effects. Note that an average of 5.6 “trains” of sequential of monomers of average length 9.6 segments adsorbed on the surface from chains of length 85. Given that each unit of the MC chain adsorbed to the surface with energy of 1.0 k_B_T, the speed of desorption of these chains is rather surprising since the energy cost of desorbing so many monomers ought to be extraordinarily high, naively an exponential of the number of adsorbed monomers. It seems likely that competition for adsorption sites by incoming chains made the desorption rate many orders of magnitude faster than it would have been for an isolated chain on the surface. The relatively fast hopping of isolated chains in the experiments of Skaug et al. [1], thus remains unexplained, or incompletely explained, by simulations such as these. Dutta, Dorfman, and Kumar [6] simulated desorption of isolated Kremer–Gest bead-spring chains in a shearing flow, but found that in the absence of flow, the chains did not desorb during the simulation, so the kinetics of desorption without flow could not be measured in this study, limiting its relevance to our work. 

Källrot and Linse (2007) studied desorption of polymers which had adsorbed under an attractive potential and were then switched to a repulsive potential, but these results are of limited use for understanding desorption from an attractive wall. Paturej, Milchev, Rostiashvili, and Vilgis (2012) studied the detachment time of polymers under tension, with a pulling force generated by viscous drag on the string of tensile blobs extending from the surface, countered by the restoring force from the blob at the end of the string in contact with the surface. They developed a theoretical prediction giving a detachment time that scales as *N^2^* for strong adsorption and overdamped dynamics, and their Monte Carlo simulation results yielded a scaling exponent on *N* of 1.96 ± 0.03, while their Langevin-like simulation which they describe as underdamped, yielded a scaling exponent of 1.75 ± 0.1 [9]. In a later study (2014) which included hydrodynamic interactions (HI) among polymer beads via dissipative particle dynamics, they found HI did not have a significant effect on the forced desorption process [10].

Mökkönen, et al. (2015) calculated the rate for a one-dimensional (1D) ideal bead-spring polymer to translocate from one basin to another in a symmetric, quartic (i.e., given by a fourth order polynomial) double-well potential [11]. After using the “nudged elastic band method” to find the saddle point as the maximum energy point along the minimum energy path, they used harmonic transition state theory with corrections and found close agreement of their predictions with brute-force Brownian dynamics and Langevin dynamics simulation. Mökkönen, et al. (2016) further improved their calculation method with a re-crossing correction and found it was more computationally efficient than forward flux sampling for achieving an equal degree of uncertainty in the rate calculation. Park and Sung showed that in such a double-well system, the activation energy reaches a plateau when *N* > *N_C_* where *N_C_* is a critical polymer size above which the transition state ceases to be a coil and is instead a stretched chain reaching from basin to basin [12,13]. These results for escape from one half of the double-well potential may have implications for polymer escape from highly convex surfaces (e.g., a nanoparticle) whose curvature leads to a maximum free energy followed by a decreasing free energy with increasing radial distance far from the surface, rather than a plateau as in desorption from a planar surface. For desorption from a flat surface, because the free energy should reach a plateau for the desorbed state, there would be no such *N_C_* such that a polymer with *N* > *N_C_* can span two thermodynamically favorable basins.

This review exposes a fundamental gap in the literature, as no published work analyzes the most basic problem of desorption of isolated chains from a simple flat surface in the absence of flow. In recent years, forward flux sampling has drastically progressed due to a repeatable workflow, innovations to reduce system constraints, and the application of the method to new fields [14]. In addition, FFS simulations use an unbiased potential and can address both equilibrium and non-equilibrium systems. Many groups have used FFS for simulating nucleation, magnetic switching, gene switching, protein or DNA behavior, and membrane transport [14]. In the polymer field, FFS has been applied to polymer translocation [15], structural relaxation [16,17], and conformational rearrangement [18,19,20]. However, to the best of our knowledge FFS has not yet been applied to polymer desorption from a surface. We aim to fill this gap using forward flux sampling. Since this is a rare event with a high energy barrier it is well suited for FFS. 

## 2. Methods 

Forward flux sampling computes the rate of transition from state A to state B [21,22]. The computation has three essential steps:

1. Sample “equilibrium”-distributed configurations from starting basin A.
2. Compute the rate at which these configurations enter the initial transitional state $L_{0}$.
3. Compute the probability that paths entering state $L_{0}$ will also enter B before returning to A.

**Figure 1:**
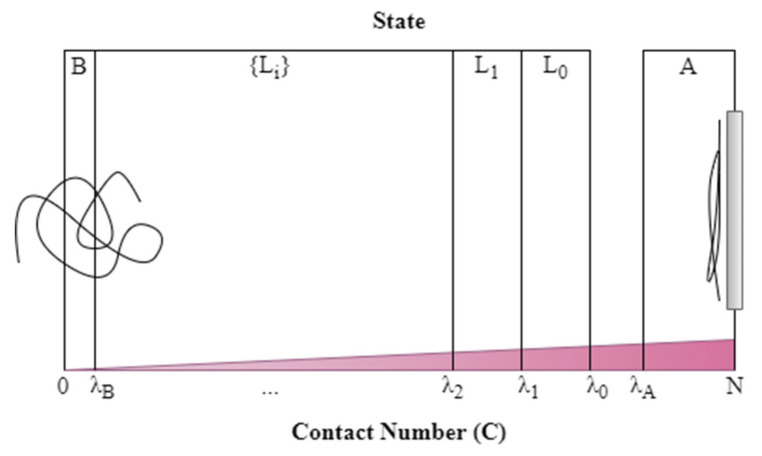
Definition of states A, $L_{0}$, $L_{1}$, …, B (shown by boxes) over the range of contact numbers between 0 and *N*. State A represents the adsorbed polymer and state B the desorbed polymer. Intermediate transitional states are named $L_{i}$. State *A* encompasses contact numbers from *N* down to 10 or 0.1*N* (whichever is smallest).

The final transition rate from state *A* to state *B* computed in an FFS simulation is a product of a “base rate” $r\left(A\to L_{0}\right)$ of configurations “transitioning” from the starting basin *A* across an interface “0” into state *L*_0_, times a product of probabilities, one for each transition from interface *i* to *i*+1. Each interface *i* will be defined by a prescribed number of “contacts” $\lambda_{i}$, where the number of “contacts” is defined later. A “state” *i* is defined by having numbers of contacts between $\lambda_{i}$ and $\lambda_{i+1}$, and basins *A* and *B* are defined by having contact numbers respectively greater than (for *A*), and less than (for *B*), a prescribed number to be defined below for *A* and for *B*. For the probabilities of transition, we only need a “yes” or “no” answer to the question whether a chain has made the transition before collapsing back to basin *A*. For the “base flux,” however, we cannot count within the “rate” every vibration of a configuration across a thin interface, since this will allow highly correlated configurations to count many times towards the transition rate. Therefore, we must create a gap over which the configuration must jump for it to count as a “transition” from basin *A* across the “initial” interface into state *L*_0_; see Figure 1. The gap has to be large enough that after the configuration has retreated back across interface 0 towards basin *A*, it must retreat far enough for the chain configuration to decorrelate before another crossing of interface_0 can count in the net rate. In the limit where the gap becomes very large, the jump crosses all the way from *A* to *B* and the crossing rate becomes the final rate of transition. If this could be accomplished within a reasonable simulation time, there would be no need for FFS. What is sought, therefore, is that the gap be made big enough that the distribution of transition times from *A* to 0 is approximately uncorrelated, and hence be exponentially distributed, while keeping it small enough to make the simulations affordable. 

With this explanation of the overall rationale, we now define how we accomplish Steps 1, 2, and 3 above. 

### 2.1. Step 1: Sample Equilibrium-Distributed Configurations from A

We first generated polymer coil configurations for a chain of *N* monomers in the absence of the wall by a random series of torsional rotations followed by simulation for sufficient time to measure the end-to-end distance autocorrelation time $\tau_{\mathrm{ends}}$. We then continued the simulation and sample one configuration after each time period $2\tau_{\mathrm{ends}}$. Each of these configurations represented an independent equilibrium configuration of the chain. Since our simulations were carried out in the overdamped limit, no momentum sampling was required, as rapid sampling of the Maxwell–Boltzmann distribution was carried out during the simulation. 

As discussed in more detail later, we then translated each equilibrium coil to a position close to the wall and simulated each for 100 N picoseconds. Chains that had contact number greater than either 10 or 0.1*N* (whichever is smallest) at this point were kept as adsorbed, where “contact number” is the number of beads “contacting” the wall, as defined precisely later, in Equation (14). Some chains whose contact number fell below the threshold were simulated for an additional pre-initialization time to obtain a contact number exceeding the threshold. The wall-contacting configurations at this point were taken as an approximation to “equilibrium-distributed” sample configurations from A. Our reaction coordinate will be defined as the contact number C, which decreases as the chain desorbs from the surface, and can be used to define discrete levels of desorption as illustrated in Figure 1.

### 2.2. Step 2: Compute the Rate at Which the Configurations from A Cross Interface 0 and Enter the Initial Transitional State $L_{0}$

Before the rate $r\left(A\to \lambda_{0}\right)$ of $A\to \lambda_{0}$ can be computed, we need to programmatically define the minimum contact number $\lambda_{A}$ defining state A and the maximum contact number $\lambda_{0}$ for the configuration to belong to state $L_{0}$. There are two closely related pitfalls we wish to avoid when setting $\lambda_{A}$ and $\lambda_{0}$.

1. Avoid counting multiple entrances into $L_{0}$ that are actually correlated vibrations around $\lambda_{0}$.
2. Avoid counting only entrances into $L_{0}$ by a few privileged chains rather than all transitioning chains, to avoid bias by exclusion.

To avoid pitfall 1, we create a gap between $\lambda_{0}$ and $\lambda_{A}$ (described below), and to avoid pitfall 2, we also require that all adsorbed chains in the basin *A* cross $\lambda_{0}$ at least once during Step 2, the initial rate-measurement step. A set R of at least 100 simulation runs, each with an independently adsorbed chain is simulated. If any of these chains, which start in A, fail to cross $\lambda_{0}$, the rate-measurement time $t_{\mathrm{rate}}$ for all these chains in set R is doubled. This process is repeated until all chains cross $\lambda_{0}$.

We assigned $\lambda_{A}$ and $\lambda_{0}$ as follows. Firstly, the time-averaged contact number (which will be defined precisely later, in Equation (14)) is calculated for a given chain simulation *i*, or run *i*, by averaging over all time steps *j* = 1,2,…:

(4) $$\langle C\rangle_{i}\equiv \frac{1}{T}\overset{T}{\underset{j=1}{\sum }}C(X_{i}\left(t_{j}\right))$$

The minimum contact number is also calculated over the time of each chain’s run:

(5) $$C_{i,\min}=\underset{j}{\min}\;C(X_{i}\left(t_{j}\right))$$

We wish $\lambda_{A}$ to represent the “outer edge” of basin *A*, and it should then be just small enough that most adsorbed chains, most of the time, have contact numbers above $\lambda_{A}$. We also wish $\lambda_{0}$ to definitely lie below the typical value for chains in basin *A* but large enough that, with a long enough simulation, all chains will eventually reach it. To obtain these two quantities $\lambda_{A}$ and $\lambda_{0}$ that satisfy these requirements, we find that a robust procedure is to first take $\lambda_{0}$ as the average of two quantities: 1) the minimum over the set R of the simulation-averaged contact number for each chain’s run, and 2) the median over R of the simulation minimum over each run; i.e.,

(6) $$\lambda_{0}\equiv \frac{\underset{i\in R}{\min}\left(\langle C\rangle_{i}\right)+\mathrm{median}\left(\left\{C_{i,\min}\;:\;i\in R\right\}\right)}{2}$$

We then assign $\lambda_{A}$ by averaging $\lambda_{0}$ and the minimum of the sample averages, which makes $\lambda_{A}$ somewhat larger than $\lambda_{0}$:

(7) $$\lambda_{A}\equiv \frac{\lambda_{0}+\underset{i\in R}{\min}\left(\langle C\rangle_{i}\right)}{2}=\frac{3}{4}\underset{i\in R}{\min}\left(\langle C\rangle_{i}\right)+\frac{1}{4}\mathrm{median}\left(\left\{C_{i,\min}\;:\;i\in R\right\}\right)$$

In order to avoid counting vibrations around $\lambda_{0}$ as “crossings” of $\lambda_{0}$, forward crossings of $\lambda_{0}$ are counted only once per forward crossing of $\lambda_{A}$. In a simulation, after the contact number drops below $\lambda_{A}$, (which constitutes a forward crossing of $\lambda_{A}$ towards $\lambda_{0}$, since forward progress is achieved by reducing the contact number λ), only the next time the contact number further drops below $\lambda_{0}$, is a forward crossing of $\lambda_{0}$ counted. For an additional forward crossing of $\lambda_{0}$ to be counted again within the same run, the contact number must first rise again above $\lambda_{A}$ and then fall again below above $\lambda_{A}$. This eliminates the vibration problem of pitfall 1.

If each simulation i∈R has at least one forward crossing of $\lambda_{0}$, then $\lambda_{A}$ and $\lambda_{0}$ are determined using the method described above and the rate of crossing of $\lambda_{0}$ is computed by dividing the total number of forward crossings of $\lambda_{0}$ by the total simulated time, excluding any time spent in *B* and time spent transitioning from *B* to *A*. (This exclusion is only important for weakly adsorbed chains that desorb spontaneously within a single simulation.) If this condition is not met, and one or more simulations fails to cross $\lambda_{0}$, then, as mentioned above, the run duration $t_{\mathrm{rate}}$ is doubled, and this whole process is repeated.

### 2.3. Step 3: Compute the Probability That Paths Entering $L_{0}$ Will Also Enter B Before Returning to A

The trick of forward flux sampling is to overcome the improbability of the $\lambda_{0}\to B$ transition by decomposing it into a sequence of more probable steps $\lambda_{0}\to \lambda_{1}\to \cdots \lambda_{n}\to B$. The boundary of each level between $\lambda_{0}$ and $\lambda_{B}$ is determined adaptively (i.e., on the fly) to achieve a target advancing probability. We launched 100 trial simulations by picking random configurations from the forward crossings at $\lambda_{0}$ that ran until they reached either $\lambda_{A}$ or $\lambda_{B}$. (For configurations chosen from forward crossings at $\lambda_{0}$, the simulations would invariably end at $\lambda_{A}$, but in subsequent simulations starting from forward crossings at $\lambda_{i}$, the probability of reaching B increases.) Each simulation has a minimum contact number; by positioning $\lambda_{1}$ at the 10th percentile of these 100 contact number minima, we ensured that approximately 10% of attempted transitions $\lambda_{0}\to \lambda_{1}$ were successful. With $\lambda_{1}$ positioned, we would run at least 1000 simulations to attain a more accurate estimate of $P\left(\lambda_{0}\to \lambda_{1}\right)$.

The process in the preceding paragraph is repeated for the $\lambda_{1}\to \lambda_{2}$ transition and so on, iteratively, until the probability of advancing to B exceeds 10%. The *B* or end basin in every case was defined as $C<\lambda_{n}=10^{-5}$. At this point $P\left(\lambda_{n}\to B\right)$ can be measured in the same way as the preceding levels. The rate for the complete process A→B is then constructed by multiplying the rate from step 2 with the product of probabilities from step 3:

(8) $$r\left(A\to B\right)=r\left(A\to \lambda_{0}\right)\overset{n-1}{\underset{i=0}{\prod }}P\left(\lambda_{i}\to \lambda_{i+1}\right)P\left(\lambda_{n}\to \lambda_{B}\right)$$

The number of levels required to measure desorption varies depending on how unlikely it is for an *A*→*L*_0_ transition to become an *A*→*B* transition. Typically, in our simulations, the number of levels varied between 10 and 100. If *A*→*L*_0_ occurs once every nanosecond for chains starting in *A*, and FFS determines that *A*→*B* takes a second, then the probability of converting *A*→*L*_0_ into *A*→*B* is 10^−9^. Given a 10% advancing probability for each level, this implies that 9 levels are required to reach *B*. 

For all intermediate levels $\lambda_{i}$ with 1 < *i* < *n*, the levels were set on the fly as described in the following paragraph. Aside from these initial runs being used to position $\lambda_{0}$ and $\lambda_{1}$, the initial rate was measured as the number of forward crossings of $\lambda_{1}$ with history traceable to $\lambda_{0}$ more recently than previous crossing of $\lambda_{1}$, summed over all 100 initial runs, and divided by the total time of the 100 runs excluding any time spent in *B* and time spent transitioning from *B* to *A*.

Randomly selecting snapshots to run at each level can exacerbate the problem of “genetic drift” by randomly denying some snapshots any attempts. For example, if 1500 simulations were launched by randomly selecting from 500 configurations, then the probability that a particular configuration is not selected even once is 499/500 to the power of 1500, giving around 0.05. Thus 25 configurations out of the 500 at each level would be randomly wiped out because they are passed over, on average. An alternative selection method that reduces genetic drift due to random selection is called fixed selection in which each snapshot at a level is selected for the same number of simulations, except for any remainder after division. The remainder is allocated among the snapshots at random. Fixed selection is more equitable in assigning attempts, and it can be used without introducing any bias [23]. We know of no downside to using fixed selection, and we believe it should always be preferred to random selection. We note that fixed selection does not eliminate genetic drift entirely, because the number of successful attempts will still fluctuate randomly, but genetic drift is reduced relative to random selection.

### 2.4. Error Analysis Accounting for Correlations

We can identify the number of uncorrelated estimates of $p_{i}\equiv P\left(\lambda_{i}\to \lambda_{i+1}\right)$ at each level and use the standard error of the mean to quantify the uncertainty in our estimate for $p_{i}$. To estimate the standard error of the mean of $p_{i}$ we must account for correlations among the runs. 

**Figure 2:**
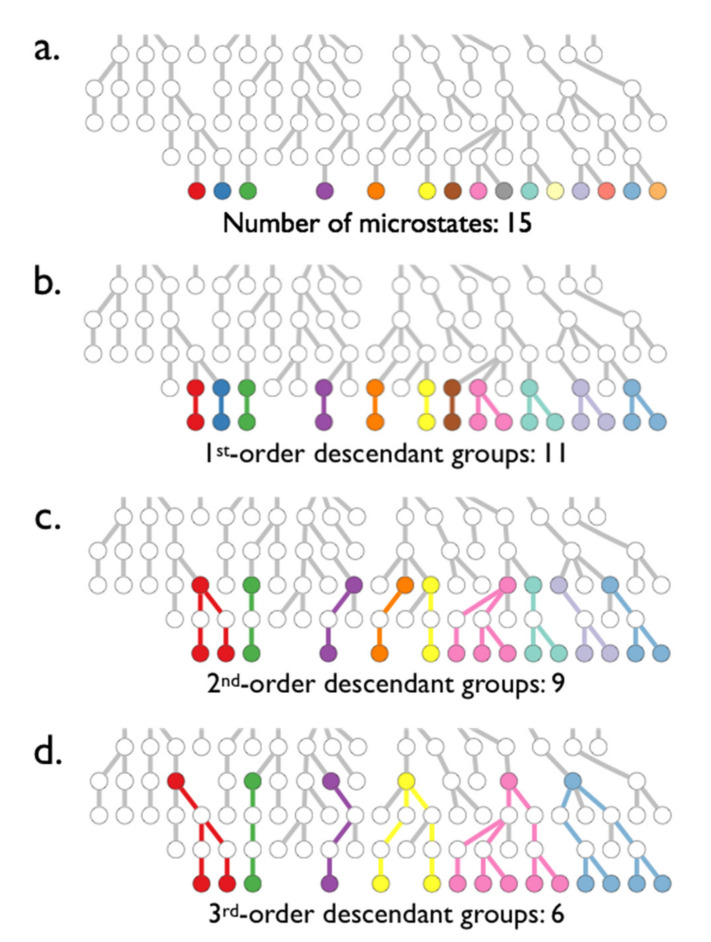
Snapshots (circles) are colored in the bottom row of each sub-figure by descendant group $G_{i}^{\left(n\right)}$, where for (**a**) n = 0 (**b**) n = 1, (**c**) n = 2, (**d**) n = 3. Each row represents a level, and each circle corresponds to an observed snapshot at the level. The common *n*th-order ancestor snapshot of each group is also colored, and the lineage paths are colored as well. Parts (**b**–**d**) show the 1st, 2nd, and 3rd-order descendant groups, respectively.

To do so, first we define descendant groups. At each level *i*, forward flux sampling yields a set of chain configurations or “snapshots”, one for each run that has reached this level from level *i* − 1. These are the configurations that will be replicated and used as starting states for the next set of simulations launched towards reaching level *i*+1. By looking back *n* levels, we can group together all snapshots at $\lambda_{i}$ that have a common ancestor at $\lambda_{i-n}$. The group for *n* = 1 contains siblings only. The group for *n* > 1 contains siblings and cousins up to their *(n −* 1)th cousins. This is illustrated in Figure 2. We use such groups of descendants at each level, which we henceforth refer to as *n*th-order descendant groups, $G_{i}^{\left(n\right)}$ to check for intragroup correlations relative to the entire set $M_{i}$ of snapshots at level *i*.

We next define $P_{i}^{\left(n\right)}$ as the set of all pairs of intragroup snapshots for the *n*th-order descendant groups at level *i*. If an *n*th-order descendant group has *k* members, then that group has *k*(*k* − 1)/2 pairs of members, and $P_{i}^{\left(n\right)}$ is the set of these pairs, along with the pairs from all other descendant groups at level *i*. Thus, the number of intragroup pairs among the six descendent groups in the bottom level of Figure 2d, added up from left to right, is 1 + 0 + 0 + 1 + 10 + 6 = 18. (A group with one member has no pairs.) We define *p_i,j_* as the fraction of copies of snapshot j that reach from level *i* to *i* + 1.

We now define the measure of intragroup correlation for the descendant groups. The descendant groups may be considered “correlated” for values of *n* up to a limit at which the correlation falls below a threshold, which we take to be e^−1^. To measure the intragroup correlation, we adapt Fisher’s intraclass correlation coefficient [24]. Then, we define $p_{i}^{\left(n\right)}$ to be the average advancing probability for level *i* corrected to remove over-averaging of configurations correlated through common ancestry, using the following equation:

(9) $$p_{i}^{\left(n\right)}=\frac{1}{\left|G_{i}^{\left(n\right)}\right|}\overset{}{\underset{g\in G_{i}^{\left(n\right)}}{\sum }}\frac{1}{\left|g\right|}\overset{}{\underset{j\in g}{\sum }}p_{i,j}$$

where the metric “|X|” is the number of elements of the set, which in the above is either set g or $G_{i}^{\left(n\right)}$. The inner sum, which is divided by the number of members $\left|g\right|$ in the descendant group of snapshots g reduces the contributions of multiple descendants of a common ancestor to just one averaged contribution. We can now calculate the intragroup correlation $r_{i}^{\left(n\right)}$ in *p_i,j_* for the *n*th-order descendant groups as:

(10) $$r_{i}^{\left(n\right)}=\frac{\left|M_{i}\right|}{\left|P_{i}^{\left(n\right)}\right|}\frac{\sum_{\left(j,k\right)\in P_{i}^{\left(n\right)}}\left(p_{i,j}-p_{i}^{\left(n\right)}\right)\left(p_{i,k}-p_{i}^{\left(n\right)}\right)}{\sum_{j\in M_{i}}{\left(p_{i,j}-p_{i}^{\left(n\right)}\right)}^{2}}$$

We can identify the smallest uncorrelated descendant groups $G_{i}^{\left(L\right)}$ as the *L*th-order descendant groups at level *i* for which:

(11) $$L=\min\left\{n\;|\;r_{i}^{\left(n\right)}\leq \frac{1}{e}\right\}$$

The standard error of the mean to quantify the uncertainty in our estimate for $p_{i}$ is now taken as:

(12) $$\sigma_{p_{i}}=\sqrt{\frac{1}{\left|G_{i}^{\left(L\right)}\right|\left(\left|G_{i}^{\left(L\right)}\right|-1\right)}\overset{}{\underset{g\in G_{i}^{\left(L\right)}}{\sum }}{\left(p_{i}^{\left(L\right)}-\frac{1}{\left|g\right|}\overset{}{\underset{j\in g}{\sum }}p_{i,j}\right)}^{2}}$$

A simpler and more conservative approach than the one just described is to assume that any common ancestor leads to correlation between descendants. Both types of error bars will be shown later as 95% confidence intervals. The thick error bars in those figures are based on the standard error of the mean with the number of independent estimates of $p_{i}$ at each level based on the smallest uncorrelated descendant groups. The thin error bars are based on the more conservative standard error of the mean with number of independent estimates of $p_{i}$ at each level based on the number of groups which shared no common ancestor.

### 2.5. Contact Number and Polymer-Wall Potential

Next, we define the adsorbed state, the interaction potential, and other simulation parameters. Later, we also describe the algorithmic details of simulation setup and analysis.

**Figure 3:**
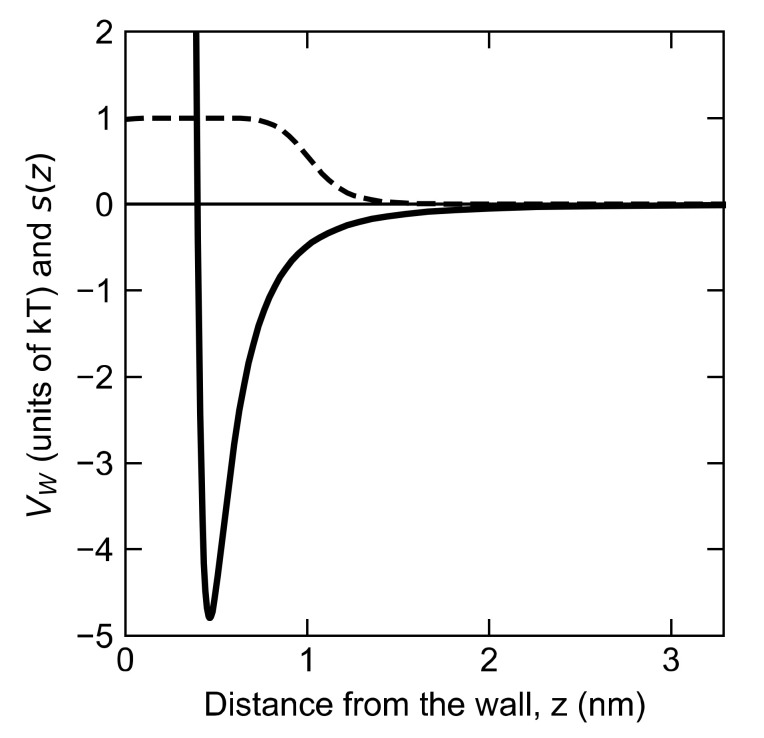
Switching function $s\left(z\right)$ (dashed line) given by Equation (13) that weights the degree to which a monomer counts as “adsorbed,” thereby adding to contact number. Monomer-wall potential (thick solid line) $V_{W}$ given by Equation (15) for $\varepsilon_{\mathrm{PW}}=1$ k_B_T. Zero potential is plotted as the thin, horizontal line.

Our adsorbed state is defined by the wall contact number C, which is defined by a continuous switching function of the distance z from the wall

(13) $$s\left(z\right)=\frac{1-{\left(\frac{z-d_{0}}{r_{0}}\right)}^{n}}{1-{\left(\frac{z-d_{0}}{r_{0}}\right)}^{m}}$$

where we take $d_{0}=0.35$ nm, $r_{0}=$ 0.7 nm, n=6, m=14. The parameter m must be larger than n for the function s(z) to have a plateau. n is made large enough to yield a smooth plateau; $d_{0}$ and $r_{0}$ are chosen to control the onset point and the length of the plateau, and m is made large enough to yield a rapid cutoff. These parameters produce the function given by the dashed line in Figure 3 that switches smoothly, but rapidly, from 1 to 0. (We calculated s using the Plumed plugin and inputting the switching function as that of rational type.) Then the contact number is,

(14) $$C=\overset{N}{\underset{i=1}{\sum }}s\left(z_{i}\right)$$

where $z_{i}$ is the z component of the position of bead i, and *N* is the number of beads in the chain. We define the desorbed state as $C\leq 10^{-5}$. This ensures the polymer is at a sufficient distance that it has had time to reach a random coil configuration upon reaching the final state, rather than remaining deformed due to its prior adsorption to the wall. To model the wall, we use the Steele 10-4-3 potential, which has been used previously for simulation of polymer at a fluid-solid interface [6].

(15) $$V_{W}\left(z;\sigma_{PW},\varepsilon_{PW}\right)=2\pi \varepsilon_{PW}\left[\frac{2}{5}{\left(\frac{\sigma_{PW}}{z}\right)}^{10}-{\left(\frac{\sigma_{PW}}{z}\right)}^{4}-\frac{\sqrt{2}\sigma_{PW}^{3}}{3{\left(z+\left(\frac{0.61}{\sqrt{2}}\right)\sigma_{PW}\right)}^{3}}\right]$$

where *z* is the distance of a bead from the wall, and $\sigma_{PW}$ and $\varepsilon_{PW}$ are the bead–wall interaction parameters.

The criterion for desorption that C drop below some pre-defined value such as $C\leq 10^{-5}$ seems reasonable, except that it has a degree of arbitrariness to it. While we expect trends to remain similar if this value is changed, precise desorption times will depend on this choice. However, even in an experiment, one must define when a chain is “adsorbed” and when it is not. How far should monomers be from the surface before the chain counts as desorbed? Desorbed monomers that are close to the surface may re-adsorb, and the polymer may drift from the surface, but then drift back towards it. Experimentally, the desorption criterion should be based on some measurement condition, such as spatial resolution of the measured distance from the surface, or some means of removing chains that get far enough from the surface to prevent re-adsorption. Ultimately, the choice of desorbed state would need to be guided by some specific experimental criterion, which would allow a more precise comparison between the calculations and measurements. Here, we content ourselves with assessing the enormous range of desorption times obtained as chain length and monomer adsorption strength are varied, and leave consideration of the much weaker dependence of desorption time on the precise boundary between adsorbed and desorbed states.

### 2.6. Langevin Equation of Motion and Polymer Models

We ran the simulations in LAMMPS at a temperature of 300 K and using a time step of 40 fs. Polymer configurations were evolved using the Langevin equation of motion:

(16) $$m{\ddot{x}}_{i}=\nabla_{x_{i}}V-\frac{m}{t_{\mathrm{damp}}}{\dot{x}}_{i}+R$$

with a damping parameter $t_{\mathrm{damp}}$ of 250 fs and a bead mass m of 45 g/mole (converted to mass per particle using Avogadro’s number). The choice of $t_{\mathrm{damp}}$ was made to keep the results in the over-damped limit, as discussed later. The bead mass m of 45 g/mole was a slight typographical error in the simulation, since for specificity we are simulating poly(ethylene oxide), or PEO, whose repeat unit has a mass of 44 g/mole, but the mass is irrelevant except for its role in setting the diffusivity, since we verified that we were in a non-inertial dynamics regime (see Figures S1 and S2). The acceleration and velocity of bead *i* are ${\ddot{x}}_{i}$ and ${\dot{x}}_{i}$, respectively. The random force ***R*** is computed by the Langevin thermostat in LAMMPS. The conservative force on particle *i*, namely $\nabla_{x_{i}}V$, is a combination of bead–bead interactions, bead–wall interactions, and bond stretch, bending, and torsion-like potentials, and is given by:

(17) $$V=\overset{}{\underset{i\neq j}{\sum }}V_{LJ}\left(r_{i,j},\sigma_{PP},\epsilon_{PP}\right)+\overset{N}{\underset{i=1}{\sum }}V_{W}\left(z_{i};\sigma_{PW},\;\epsilon_{PW}\right)+\overset{N-1}{\underset{i=1}{\sum }}k_{b}{\left(r_{i,i+1}-l_{b}\right)}^{2}\;+\overset{N-2}{\underset{i=1}{\sum }}k_{A}{\left(\theta_{i,i+1,i+2}-\theta_{A}\right)}^{2}+\overset{N-3}{\underset{i=1}{\sum }}k_{D}{\left(r_{i,i+3}-r_{D}\right)}^{2}$$

where $\sigma_{PP}$, $\epsilon_{PP}$, $\sigma_{PW}$, $\epsilon_{PW}$, $k_{b}$, $l_{b}$, $\theta_{A}$, $k_{D}$, and $r_{D}$ are model parameters, $r_{i,j}$ is the distance between bead *i* and bead *j*, and $\theta_{i,j,k}$ is the bending angle formed by beads *i*, *j*, *k*. *V_LJ_* is the standard Lennard Jones interaction potential between beads. *N* is the number of beads in the chain. The parameter values for the chain models considered are in Table 1. Unless otherwise noted, the bond length is $l_{b}=0.33\;\mathrm{nm}$ and bond stretching constant $k_{b}=16000\;\mathrm{kJ}/\mathrm{mole}$. In cases without excluded volume between beads, $\epsilon_{PP}=0$. In cases with excluded volume, $\epsilon_{PP}=0.2\;\mathrm{kJ}/\mathrm{mole}$ and $\sigma_{PP}=0.43\;\mathrm{nm}$. For freely-jointed chains (FJC), $k_{A}=0$, whereas for freely-rotating chains (FRC), $k_{A}=2000$ kJ mole^−1^ radian^−1^ and $\theta_{A}$ = 111.58°. $\theta_{A}$ was chosen to yield a persistence length of 1 nm for an ideal freely-rotating chain. $\sigma_{PP}$, $\epsilon_{PP}$, and $l_{b}$ were adopted from a coarse-grained model for PEO, and $k_{b}$ and $k_{A}$ were chosen to approximate stiffness while retaining numerical stability. The Lennard Jones potential was used only when the excluded volume (EV) interactions were present, and torsional potential (T) was only used for chains with torsion.

We verified that the Lennard-Jones excluded volume interaction was sufficient to prevent chains from crossing each other. The parameters except $\sigma_{PP}$ and $\varepsilon_{PP}$ were borrowed from a Dry Martini model for PEO which had the correct dependence of gyration radius on *N* for a PEO polymer [25].

The mobility of an isolated bead is given by the inverse friction coefficient $\mu_{1}=\zeta^{-1}=t_{\mathrm{damp}}/m$. Using the Einstein relation, the monomer diffusivity is given by $D_{1}=\mu_{1}k_{B}T=\left(t_{\mathrm{damp}}/m\right)k_{B}T$, which (using $t_{\mathrm{damp}}$ of 250 fs and m of 45 g/mole given earlier, as well as Avogadro’s number) is around 10^−4^ cm^2^/s, and is around a decade higher than physically expected, but accomplishes our goal of achieving the over-damped limit. At low ζ, the molecule’s momentum will decay slowly, and the molecule may oscillate within a potential well. At high ζ, the molecule’s dynamics may be overly sluggish, and simulation performance suffers needlessly. Klimov and Thirumalai found that the folding rate in simulations of certain small polypeptides increases linearly with friction coefficient ζ at low ζ, reaches a maximum at intermediate ζ, and decreases as ζ^−1^ at high ζ [26]. The decrease in folding rate with increasing ζ at high ζ is an intuitive result of increasing friction in overdamped dynamics, whereas the less intuitive increase of folding rate with increasing ζ at low ζ may be attributable to the increasing frequency of thermal fluctuations with increasing ζ. Slow thermal dissipation of momentum implies slow thermal fluctuation in momentum, and although the uninterrupted momentum of a gas particle leads to a large diffusivity of the particle, the uninterrupted momentum of chain segments evidently leads to inefficient searching of configuration space.

A primary concern of ours was to avoid results that were sensitive to small changes in bead mass m and bending constant $k_{b}.$ At coarse-grained scales in water, we expected inertial effects to be minimal, so we ensured that desorption times were in the non-inertial scaling regime, such that desorption times scaled as ζ^−1^. 

**Figure 4:**
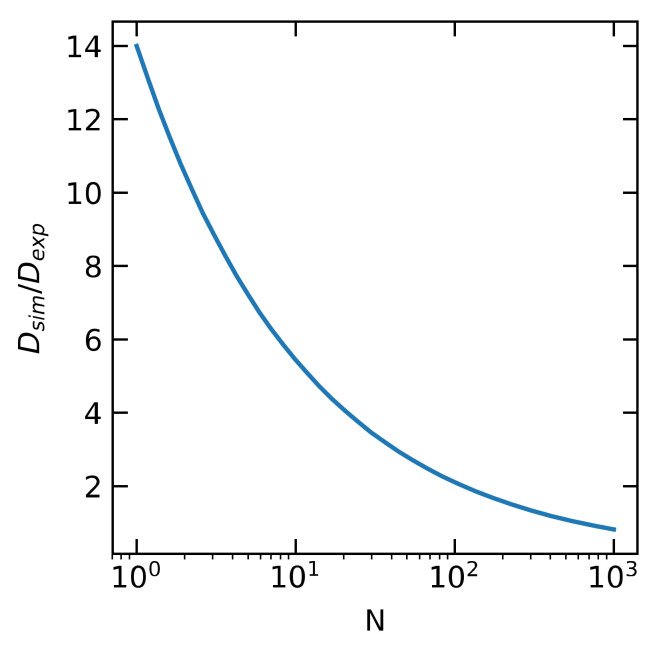
Ratio of simulation diffusion coefficient $D_{\mathrm{sim}}$ to “experimental” diffusion coefficient $D_{\exp}$ as a function of *N*, the number of monomers. This serves as the conversion factor to convert simulation time to “experimental time,” as discussed in the text.

To compare simulation times with the experimental ones, one can scale from simulation time $t_{\mathrm{sim}}$ to an experimental time $t_{\exp}$ using $t_{\exp}=t_{\mathrm{sim}}\frac{D_{\mathrm{sim}}}{D_{\exp}}$, where $D_{\mathrm{sim}}$ and $D_{\exp}$ are the self-diffusion coefficients of a polymer coil in solution from the simulation and what is expected in the experiment, respectively. Given our neglect of hydrodynamic interactions, the dependence of $D_{\mathrm{sim}}$ on *N* has Rouse ($D_{\mathrm{sim}}\propto N^{-1}$) scaling, i.e., $D_{\mathrm{sim}}\left(N\right)=\frac{D_{1}}{N}$, as opposed to the experimental $D_{\exp}\propto N^{-0.589}$ scaling expected for a dilute polymer in a good solvent. This means that the time scaling factor $\frac{D_{\mathrm{sim}}}{D_{\exp}}$ scales as $N^{-0.411}$. For a good solvent, in particular for PEO chains in water at room temperature, using $D_{\exp}\propto N^{-0.589}$, the time scaling factor is found to vary between 14.0 and 0.816 as *N* varies between N=1 and N=1000, respectively, as plotted in Figure 4. To obtain these scaling factors, in addition to the scaling law $\frac{D_{\mathrm{sim}}}{D_{\exp}}\propto N^{-0.4}$, we note that for the *N* = 50 polymer in simulation we observe $D_{\mathrm{sim}}$= 277 µm^2^/s whereas interpolating from the diffusion coefficient for PEO in water using a table in the SI of Skaug et al. we find $D_{\exp}$ = 99 µm^2^/s. This means, for the N=50 polymer, that simulation times should be increased by a factor of 2.8 (or rates should be decreased by a factor of 2.8) to compare with the experiments in Skaug et al. In the results section, we plot the dimensionless $\frac{t_{\mathrm{sim}}D_{\mathrm{sim}}}{R_{g}^{2}}$, thereby multiplying this dimensionless quantity by the real $\frac{R_{g}^{2}}{D_{\exp}}$ yields the real time $t_{\exp}$ corresponding to the simulated chain.

### 2.7. Equilibrating and Adsorbing Polymer Coils

As mentioned in Step 1 of the FFS methodology discussed above, random polymer configurations were generated for all model types in Table 1 by first randomly selecting and rotating bonds. Detailed balance was not respected in this process due to the bending potential, excluded volume potential, and pseudo-dihedral potential that are imposed on many of the simulated chains, so the set of initial configurations was not necessarily an equilibrium sample. Each configuration was then evolved in LAMMPS with Langevin dynamics, the end-to-end distance autocorrelation time $\tau_{ends}$ was calculated, and configurations were saved at intervals of 2$\tau_{ends}$, as mentioned earlier. The resulting nearly independent coil configurations were then transplanted into a simulation box with the wall potential described above, and they were translated along the z direction to bring one of their beads to a *z* position of 0.7 nm, with the remaining beads at larger z values. The configurations were then evolved using Langevin dynamics until the chain center of mass had either moved 12 nm from the wall, 90% of its beads were adsorbed, or a maximum time in ps equal to 100 N had elapsed, which proved to be an efficient method of generating adsorbed chains to be used as starting states for the FFS simulation. Once the simulation stopped, if at least 10 beads or 10% of beads were in contact with the wall (as defined by the contact number in Equations (13) and (14)), the configuration was saved as a pre-adsorbed configuration to be used in forward flux sampling of the desorption process. Tools used to carry out FFS sampling method, analysis of transition paths, and implementing the scheme in LAMMPS, are expanded on in the Supplementary Materials. 

## 3. Results

**Figure 5:**
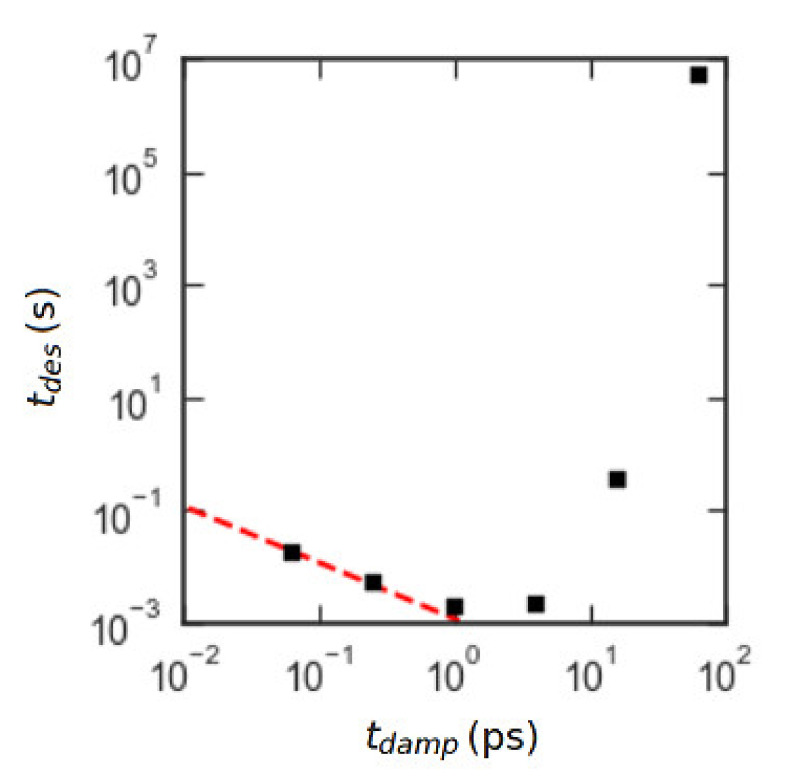
Plot of mean desorption times $t_{\mathrm{des}}$ calculated with forward flux sampling for a series of freely jointed chains (FJCs) with *N* = 50, ε_PW_ = 0.6 kJ/mol and with varying damping time $t_{\mathrm{damp}}$ for fixed particle mass of 45 gm/mol. Black squares are forward flux sampling (FFS) data, and the red dashed line has slope −1 on the log-log plot to illustrate the inverse scaling of $t_{\mathrm{des}}$ with $t_{\mathrm{damp}}$ as expected for non-inertial dynamics.

We first verified that the damping time $t_{\mathrm{damp}}=0.25\;\mathrm{ps}$ yielded non-inertial dynamics. We also ensured that the results were insensitive to the change in the bending constant $k_{b}\to k_{b}/2$ and bead mass m→m/3. The data verifying this are in the Supplementary Materials. The main result can be seen in Figure 5. The square markers are forward flux sampling results for an N=50 chain for six different values of $t_{\mathrm{damp}}$. The desorption time for $t_{\mathrm{damp}}=0.25\;\mathrm{ps}$, which is the one used in our “production” runs, is the second-from-the-left point. Comparison with the dashed line of log-log slope −1 shows that the results for $t_{\mathrm{damp}}=0.064\;\mathrm{ps}$ and $t_{\mathrm{damp}}=0.25\;\mathrm{ps}$ are in the non-inertial regime.

**Figure 6:**
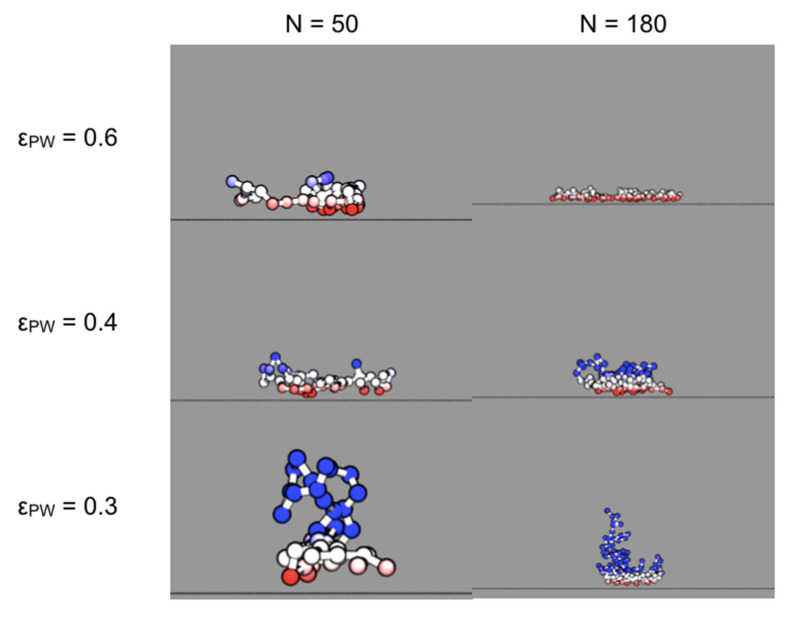
Snapshots of chains with three different polymer–wall interaction energies ε_PW_ and of two different lengths. These chains did not have excluded volume interactions. Beads are colored on a scale of red (adsorbed) to blue (desorbed), where z≤0.4 nm is red, z=0.7 nm is white, z≥1 nm is blue, and intermediate colors are blended. The units of ε_PW_ are kJ/mol.

Desorption times presented in this section are *simulation* times which have not been converted to experimental (i.e., real) times for PEO chains in a good solvent, as discussed in the earlier section, *2.6. Langevin equation of motion and polymer models*. The conversion factors for all simulations are within an order of magnitude of unity. In Figure 6, we show six snapshots of polymer chains of two different lengths (*N* = 50, 180) and three different polymer–wall interaction energies (ε_PW_ = 0.6, 0.4, 0.3), here in units of kJ/mol. (To convert to units of k_B_T at 300 K, divide these values by around 2.5.) For ε_PW_ = 0.6, the adsorbed polymer resembles a pancake, confined to the potential well adjacent to the wall. For ε_PW_ = 0.3, the polymer has considerably more configurational freedom. The configurations suggest that the mean-field approach of Equations (1)–(3) should work for polymers with $\varepsilon_{\mathrm{PW}}\geq \;0.6$.

**Figure 7:**
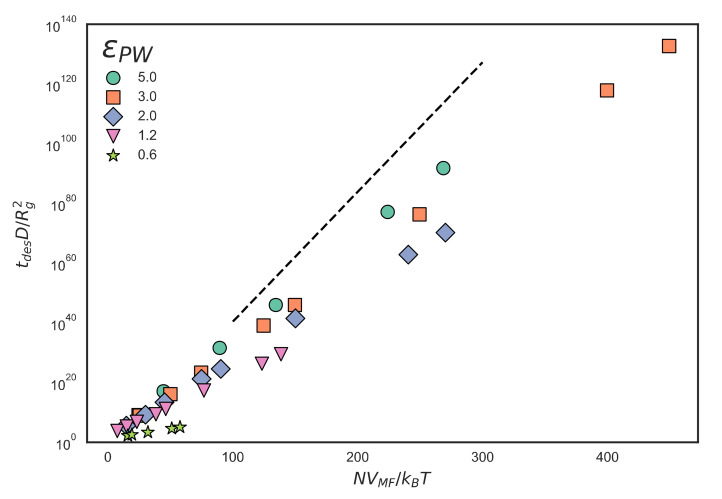
Desorption times calculated from FFS for freely-jointed chains with excluded volume (FJC+EV) polymers using several values of ε_PW_ in units of kJ/mol and plotted against $\frac{NV_{MF}}{k_{B}T}$ where $V_{MF}$ is the mean-field potential defined in Equation (1). The dashed line represents $\frac{t_{\mathrm{des}}D}{R_{g}^{2}}\propto \exp\left(\frac{NV_{MF}}{k_{B}T}\right)$ where A is an arbitrary constant selected for visual clarity and $R_{g}$ is the radius of gyration. Error bars are omitted because they are smaller than the marker size in almost all cases, because of the enormity of scale of the y-axis.

With the FFS scheme and simulation parameters verified to produce non-inertial dynamics, we first check in Figure 7 the strong mean-field scaling of dimensionless desorption time with the exponential of $NV_{MF}\left(z;\sigma ,\epsilon \right)/k_{B}T$ given by Equation (3), with *V_MF_* as defined in Equation (1). The free chain diffusivity D can be estimated as the bead diffusivity, which is around 10^−4^ cm^2^/s, divided by *N*, giving values of *D* down to as low as around 10^−6^ cm^2^/s = 10^8^ nm^2^/s for *N* = 100. The approach to strong scaling is evident as the slopes approach the dashed line with increasing ε_PW_. It is surprising that even for ε_PW_ = 5.0 kJ/mol the slope does not completely reach the strong scaling slope shown by the dashed line in Figure 7. It seems that Equation (3) is not completely valid, possibly because the mean-field equation is missing some entropic terms.

**Figure 8:**
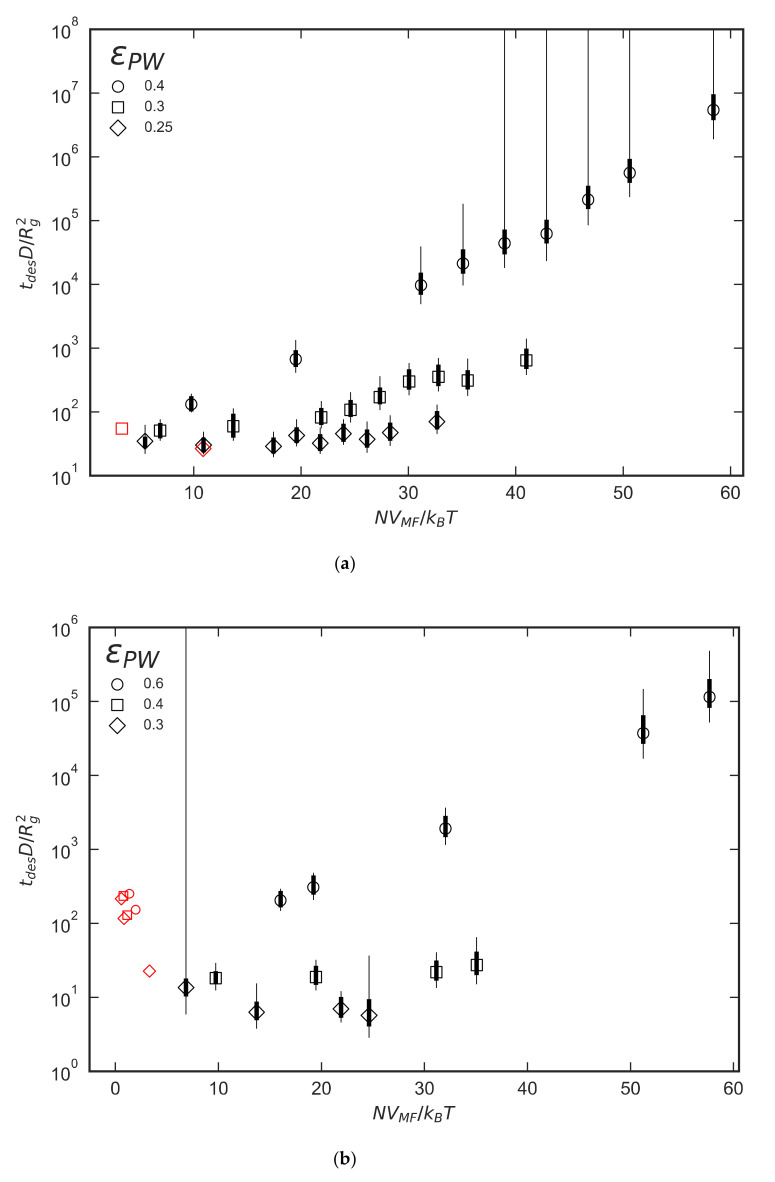
Plot of dimensionless desorption time vs. *NV_MF_* for (**a**) FJC and (**b**) FJC+EV polymers. FFS results are colored black and brute-force results are colored red. ε_PW_ is in units of kJ/mol. The thick error bars are based on Equation (12), and the thin ones are based on the conservative assumption that any common ancestor leads to perfect correlation between descendants, as discussed in the text.

Figure 8 shows the desorption times for short-chain polymers and low interactions strengths for both FJC and FJC+EV polymers. The *x*-axis again is *N* scaled by *V_MF_,* where *V_MF_* is constant for each ε_PW_ data series. After the initial small-*N* regime before the polymeric configurational penalty takes full effect, there is for ε_PW_ greater than a critical value, a crossover to an exponential dependence of desorption time on *N*, as shown by linear relations on the semilog plots in Figure 8. For the weakest-adsorbing polymers in Figure 8 the dimensionless desorption time appears to be almost independent of *N*. Due to the power-law dependencies of *R_g_* and *D*, this implies that *t*_des_ itself has a power law dependence on *N*. Whereas in the experiments of Skaug et al. [1] $t_{\mathrm{des}}\propto D^{-1}\propto N^{0.6}$, we observe $t_{\mathrm{des}}\propto R_{g}^{2}D^{-1}\propto N^{2\nu +1}$ where ν≈0.5 for the FJC without EV and ν≈0.6 for the FJC with EV. Thus, our scaling law for the weakly adsorbing limit is $t_{\mathrm{des}}\propto N^{\alpha }$, where α≈2.0−2.2, which is close to the scaling observed experimentally by Johnson et al. [3] for desorption in the presence of more strongly adsorbing chains, and by Mattice and coworkers [8] in lattice simulations of densely adsorbing unentangled chains. The cross-over from the weakly to strongly absorbing regime occurs at a value of ε_PW_ that seems to depend slightly on the details of the polymer chain, with a cross-over value of around ε_PW_ ≈ 0.3 kJ/mol for freely jointed chains without excluded volume and around ε_PW_ ≈ 0.5 kJ/mol for the same chains with excluded volume interactions. This difference is not surprising, since swelling due to excluded volume makes monomers somewhat less likely to adsorb, which can have a significant effect when adsorption is marginal. The thick and thin error bars in Figure 8 represent the two methods of estimating error discussed in Section 2.4 of the Methods section, both of which account for correlations in FFS trajectories based on common ancestry. The thick error bars allow a decay of correlation with increasing distance from the common ancestor, while the thin error bars assume that any correlation remains perfect regardless of how remote the common ancestor is, implying that chain configurations never forget that they are descended from the same common ancestor. Visual inspection of the data presented in the results section reveals limited scattering resulting in a linear trend, which suggests that the perfect correlation used to calculate the thin error bars is overly cautious. While some correlation between groups with common ancestors may exist, requiring no common ancestor for independence appears to be an unnecessarily restrictive constraint. 

**Figure 9:**
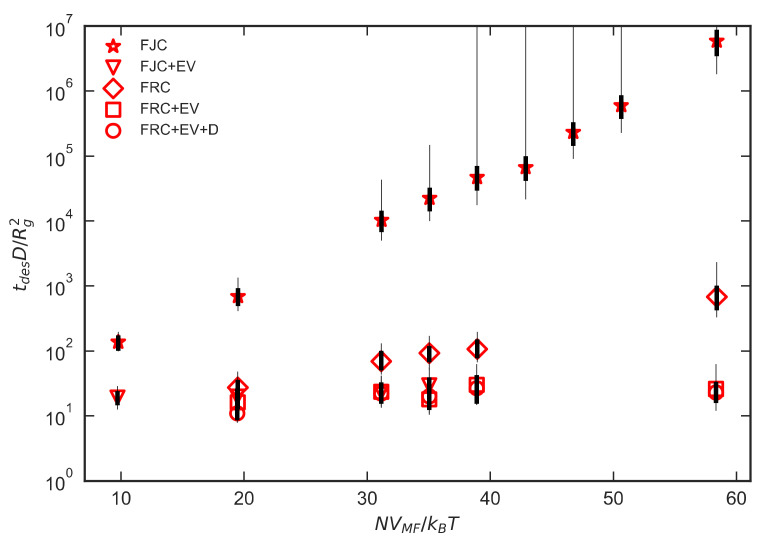
Mean desorption times for chains with $\varepsilon_{PW}=0.4$ kJ/mol and varying internal potentials. Legend labels correspond to the parameter definitions in Table 1. FJC: freely-jointed chain, FJC+EV: freely-jointed chain with excluded volume, FRC: freely-rotating chain, FRC+EV: freely-rotating chain with excluded volume, FRC+EV+D: freely-rotating chain with excluded volume and dihedral-like potential. The thick and thin error bars are discussed in the caption to Figure 8.

Figure 9 shows dimensionless desorption times on a semilog plot for chains with various internal potentials and ε_PW_ = 0.4 kJ/mol. For this value of ε_PW_, the addition of a bending potential and excluded volume reduce the scaling exponent dramatically. The data for FJC (stars) and FRC (diamonds) polymers do not follow a power law, as can be seen if they are plotted on a log-log plot (not shown) and are, therefore, evidently still exponential. For the chains with EV, the data show a weak dependence on N. The range of data would need to be extended to verify or rule out a power law dependence. While the results for the freely jointed chains show strongly adsorbing behavior, with exponential growth of desorption time with N, the less flexible freely rotating chains (which have a bond angle potential but no torsional potential) have a marginal behavior at ε_PW_ = 0.4, with a hint of a transition to the strongly adsorbing regime at the larger chain lengths. An interesting question is whether all the chains, if long enough, would eventually transition to strongly adsorbing behavior, and, if so, what sets the transition point.

## 4. Discussion

The experimental results of Skaug et al. [1] for the mean polymer desorption time $t_{\mathrm{des}}$ of a dilute chain yield two main conclusions. One is that $t_{\mathrm{des}}$ lies between 0.1 s and 1 s for *N* = 45 to *N* = 1000. In the dimensionless time units used in Figure 9, these show a 10-fold drop from between 10^4^ and 10^5^ for *N* = 45 to 10^3^ to 10^4^, for *N* = 1000. That is, Skaug’s desorption times increase less than linearly with chain length, so that, when rescaled using D/R_g_^2^, they decrease with increasing chain length. Contrary to this, we never observe in our simulations the dimensionless desorption time decrease with increasing *N*, although for weak adsorption, the dimensionless desorption time does become insensitive to chain length. It is apparent that a power-law scaling in which $t_{\mathrm{des}}$ increases from 0.1 s to 1 s over the range *N* = 45 to *N* = 1000 is not achievable within our coarse-grained models. Also, after converting the desorption times in the weak adsorption regime in Figure 8 and Figure 9 to real times, the simulated polymers desorb several orders of magnitude faster than in the experiments of Skaug et al. From Figure 8, in the weak desorption regime, the dimensionless desorption time is around 30. Since D is no smaller than around 10^8^ nm^2^/s, and R_g_^2^ is no higher than around 10 nm^2^, this corresponds to t_des_ ~1 µs rather than ~1 s, reported by Skaug et al. On the other hand, t_des_ of more concentrated chains measured by Frantz and Granick [2] scales exponentially with chain length, as predicted in our strong desorption limit, but t_des_ increases by no more than three orders of magnitude for an increase of *N* from 1000 to 10,000, implying that the prefactor of *N* in the exponential must be around 0.001, suggesting that the average binding free energy per monomer is a very small fraction of k_B_T. Thus while the desorption time measured by Skaug et al. is too long to be consistent with our “weakly adsorbing” regime, the desorption time measured by Frantz and Granick in a “strongly adsorbing” regime is too fast, and depends too weakly on *N* to be consistent with our simulations. The simulations seem clearly to be missing one or more physical features of the experiments. Below, we consider three hypothetical missing features that could explain both the different scaling laws with *N* and the different magnitudes of desorption time between experiment and simulations.

1. In the work of Skaug et al. [1], the amphiphilicity of the repeat unit may matter. Polyethylene oxide is understood to be amphiphilic on a monomer scale. This has been confirmed in at least one experiment [27] and simulation [28]. Possibly, the amphiphilic nature of the monomer should be explicitly included in the model. In other words, an atomistic chain with implicit solvent is the minimal polymer model that might yield accurate desorption times.
2. Atomistic detail of the repeat unit and water: The coarse-grained inter-bead potential in our simulation is the same in solution and at the interface. Given the hydrogen bonding or hydrophobic interactions between surface solvent molecules and adsorbed PEO or PS, the effective interaction potential between adsorbed monomers could be altered.
3. Surface heterogeneity or roughness: If the surface is unevenly hydrophobic or rough on the nanoscale, segments of the chain may become attached to difficult-to-find patches of surface. If the polymer segments, once desorbed from such patches, could not locate them again before the entire chain desorbs, then the sequential desorption mechanism for the power law desorption time scaling could be justified.

The first hypothetical explanation could be tested with a finer-grained model for PEO. Given the computational expense of FFS, an implicit solvent for the polymer is a must, but an atomistic implicit-solvent model of PEO is feasible with proper parameterization with respect to solution and adsorbed configurations, with special attention paid to the torsional angle distributions when adsorbed to the surface. The second hypothetical explanation could be investigated with a fully atomistic, albeit very expensive, simulation. The third hypothetical could, in principle, be checked in the laboratory, but a much finer time resolution (and shorter exposure) would be needed. A cursory review of recent progress in total internal reflection fluorescence microscopy (TIRFM) seems to indicate that state-of-the-art time resolution for single-molecule microscopy is at best ~10 ms [29].

## 5. Conclusions

We computed the polymer desorption times in the absence of an external field or flow for a range of coarse-grained polymer models using forward flux sampling. Our main findings are:

1. The simple strong-adsorption scaling appears to be asymptotically approached by our simulation results at least for monomer adsorption strengths greater than around 0.3 kJ/mol. This is in line with the comment by Fleer et al. that polymer adsorption under quiescent conditions is generally irreversible. They note that diffusion-controlled desorption alone is extremely slow, but the kinetic barrier to each desorption event can also be astronomical.
2. Excluded volume interactions and bending angle potentials reduce the mean desorption time and also weaken the scaling of $t_{\mathrm{des}}$ with *N*.
3. For the coarse-grained chains we studied, the scaling and its prefactor seem to be tightly coupled such that, with these models, there is no way to achieve the higher magnitude of $t_{\mathrm{des}}$ of stronger adsorbing chains with the weak *N*-dependence of the weaker-adsorbing chains that seems to have been observed in the experiments of Skaug et al. At the same time, the exponential N-dependence observed in non-dilutely adsorbed chains by Frantz and Granick is too fast and has too weak an exponential dependence to be consistent with our simulations.

We did not explore the role of persistence length in detail. One reason for this is that we do not expect the persistence length, a property of a coil in solvent, to play a direct role in the adsorbed polymer “trains” on the surface. We also emphasize that our implicit solvent model does not include hydrodynamic interactions, so our simulated polymer follows Rouse dynamics. We note that Dutta et al.^5^ found that hydrodynamic interactions between the polymer and the wall were essential to simulate the shear-induced desorption of isolated polymers, and that they are also important in desorption under quiescent conditions, but we defer such an investigation to later. 

Future computational work should investigate more closely the sensitivity of polymer desorption times to the choice of the desorption criterion. The *N*-independence of $\frac{t_{\mathrm{des}}D}{R_{g}^{2}}$ for the weakest-adsorbing chains suggests that the overall desorption rate is no longer dominated by a polymer detachment process and instead is dominated by diffusion of the chain through some distance proportional to $R_{g}$. This is at odds with the experimental findings of Skaug et al. for PEO desorption from silica surfaces coated with trimethylsiloxane (TMS), showing $t_{\mathrm{des}}D$ independent of *N.* More work should be done to understand the desorption rate in this limit and whether the $R_{g}$ dependence predicted by our simulations is reflective of the Skaug et al. experiments, whether the $R_{g}$ dependence is an artefact of our method, or whether there is an alternative mode of polymer surface diffusion that has not yet been considered, such as heightened mobility after nearly all contacts have been broken. In this work, we used a particular (small) value for the continuous contact number C defined in Equation (14) required for desorption. Although the monomer-wall contact switch function $s\left(z\right)$ defined in Equation (13) decays rapidly as the number of detached monomers increases, all monomers collectively contribute to C so that longer chains must migrate farther from the surface to satisfy the desorption criterion $C<10^{-5}$. The effect may be to increase the calculated $t_{\mathrm{des}}$ several-fold for long chains or possibly confer on $t_{\mathrm{des}}$ a dependence on a length scale proportional to $R_{g}$. The specific criterion may have an especially important effect when studying regimes in which $t_{\mathrm{des}}$ scales very weakly with *N*, as it may inflate the scaling of $t_{\mathrm{des}}$ with *N* relative to what is observed experimentally. There may also be significant performance advantages to using a hard cutoff for $s\left(z\right)$ at which monomers no longer contribute to C, because the FFS algorithm will not have to wait for long chains to migrate as far from the surface.

Another area for future study is how the monomer-scale amphiphilicity of PEO or other chains affects the desorption rate. This could be investigated with an implicit-solvent atomistic model of PEO that includes more detailed intramolecular potentials than those studied here, or perhaps a simpler model with alternating attractive and repulsive monomer–surface interactions would suffice. A key question is whether desorption times of order ~1 s (rather than ~1 μs as we predict) can be consistent with a weak scaling between $t_{\mathrm{des}}\;\mathrm{and}\;N$. It would also be worthwhile to study partially sticky copolymer chains. For weakly adsorbing chains which meander to and from the adsorbing surface, it may be possible to effectively coarse-grain the adsorbing trains into individual, sticky beads. Another excellent area for study is the role of polymer or surface heterogeneity on the desorption time and its scaling with chain length.

Future studies of more complex polymer desorption phenomena should take advantage of recent advances that modified FFS algorithms to account for time-dependent dynamics, “jumpy” dynamics, and multiple metastable states [14]. For example, a time-dependent flow field could be introduced, or the attraction between the wall and polymer may be reduced resulting in faster ‘jumps’ away from the wall, or the surface could be patterned resulting in multiple stable basins.

## Figures and Tables

**Table 1:** Parameter values for the chain models considered. N/A means not applicable—that the value was irrelevant because its potential was set to zero. A blank space means the value was varied. The double quote means the value is repeated from the cell above. N, and $\epsilon_{PW}$ are not listed in the table because they were allowed to vary for each chain model. Simulation temperature was 300 K.

	nm	kJ/mol	nm	kJ/mol/nm^2^	nm	kJ/mol/rad^2^	deg.	kJ/mol/nm^2^	nm
	$\sigma_{PP}$	$\varepsilon_{PP}$	$\sigma_{PW}$	$k_{b}$	$l_{b}$	$k_{A}$	$\theta_{A}$	$k_{D}$	$r_{D}$
FJC ^1^	N/A	0	0.47	16,000	0.33	0	N/A	0	N/A
FRC ^2^	“	“	“	“	“	2000	“	“	“
FJC+EV ^3^	0.43	0.2	“	“	“	0	“	“	“
FRC+EV	“	“	“	“	“	2000	112	“	“
FRC+EV+T ^4^	“	“	“	“	“	“	“	60	0.6

^1^ freely-jointed chains, ^2^ freely-rotating chains, ^3^ excluded volume, ^4^ torsional potential.

## Supplementary Materials

The following are available online at https://www.mdpi.com/2073-4360/12/10/2275/s1: Figure S1: Desorption times for monomers, dimers, and trimers at varying polymer–wall interaction energies and two bond spring constants, Figure S2: Desorption times for monomers, dimers, and trimers at varying polymer–wall interaction energies and two bead masses.
