# Supplementary material for: Forward Flux Sampling of Polymer Desorption Paths from a Solid Surface into Dilute Solution
Source: Polymers (Basel). 2020 Oct 3;12(10):2275. doi: 10.3390/polym12102275 (PMC7601496; doi:10.3390/polym12102275)
Supplement: Supplementary file 1 [file polymers-12-02275-s001.pdf]

Supplementary Materials for "Forward Flux Sampling of Polymer Desorption Paths from a Solid Surface into Dilute Solution"

Achieving the overdamped limit.

We here verify that dynamics are non-inertial and insensitive to the bead mass m and also insensitive to the bond spring constant k_b by testing a monomer, dimer, and trimer under varying m and k_b. In Figures S-1 and S-2, we make two tests of non-inertial dynamics for t_{damp} = 0.064 ps, 0.25 ps, and 4 ps. Here t_{damp} = m/ ζ is varied by changing the particle friction coefficient ζ, from 12 x 10^{-10} to 3 x 10^{-10} to 0.19 x 10^{-10} gm/s at fixed mass m of 45 g/mole = 7.5 x 10^{-23} gm/particle, for t_{damp} = 0.064 ps, 0.25 ps, and 4 ps, respectively. We plot the brute-force (i.e. direct Langevin simulation, averaged over many runs) desorption times (black circles) versus ε_{PW} for monomers, dimers, and trimers at each t_{damp}. In Figure S-1, in each sub-figure we use two bond spring constants, differing by a factor of 2, which does not have a systematic discrepancy on any of the three damping times tested. (The smaller markers are the results for the simulations with $k_b/2$.) For the monomers in each figure (squares), we compare the brute force desorption times with the predictions based on a simple 1D mean first passage time calculation (red X's). The brute force simulation times and the mean first passage time calculations are in good agreement for the monomers with t_{damp} = 0.064 ps and 0.25 ps but not with t_{damp} = 4 ps (the third sub-figure). This indicates the simulations with t_{damp} = 4 ps have inertial dynamics and are not in the overdamped limit. In Figure S-2, we reduce the bead mass by a factor of 3 for each value of the friction coefficient, which does not have a systematic effect for ζ = 12 x 10^{-10} and 3 x 10^{-10} as shown in the left two sub-figures, but it does have a systematic effect for ζ = 0.19 x 10^{-10} gm/s, in the right sub-figure, further confirming its inertial dynamics when the friction coefficient is too small. Finally, we check that the desorption times generated with forward flux sampling has overdamped friction with desorption proportional to the friction coefficient ζ, which is inversely proportional to t_{damp} when mass m is fixed. The expected scaling is plotted as a dashed red line in Figure 5 in the manuscript for an N = 50 chain. It is evident that t_{damp} = 0.25 ps (with mass of 45 gm/mol) lies in the strong friction regime. For all subsequent simulations, we use t_{damp} = 0.25 ps with mass of 45 gm/mol.

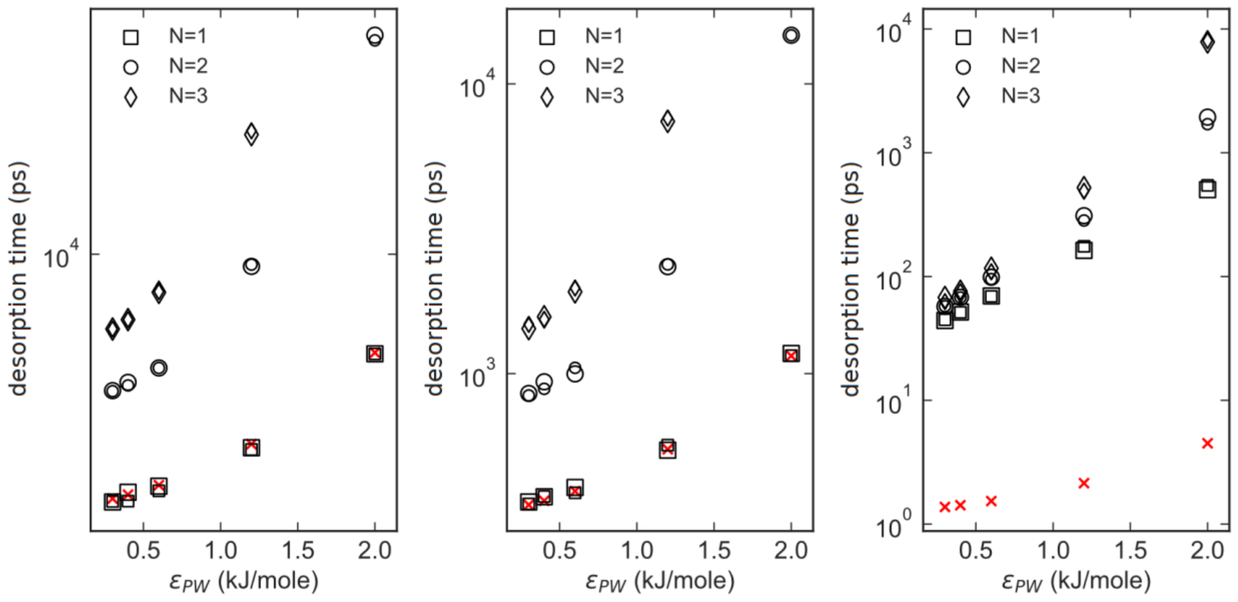

Figure S-1 Desorption times for monomers, dimers, and trimers at varying polymer-wall interaction energies and two bond spring constants. Desorption times measured by direct Langevin simulation for monomers (squares), dimers (circles) and trimers (diamonds). From left to right, t_{damp} = 0.064 ps, 0.25 ps, and 4 ps, with these differences obtained by changing particle friction as discussed in the text from ζ = 12 x 10^{-10} to 3 x 10^{-10} to 0.19 x 10^{-10} gm/s at fixed mass m = 45 gm/mol. Mean first passage times for monomers calculated with the Smoluchowski equation are plotted with red X's. Larger symbols indicate

simulations with bond spring constant 16000 kJ/mol nm^2, and smaller symbols indicate simulations with 8000 kJ/mol nm^2. In none of the cases does changing the spring constant by a factor of two lead to systematic discrepancy.

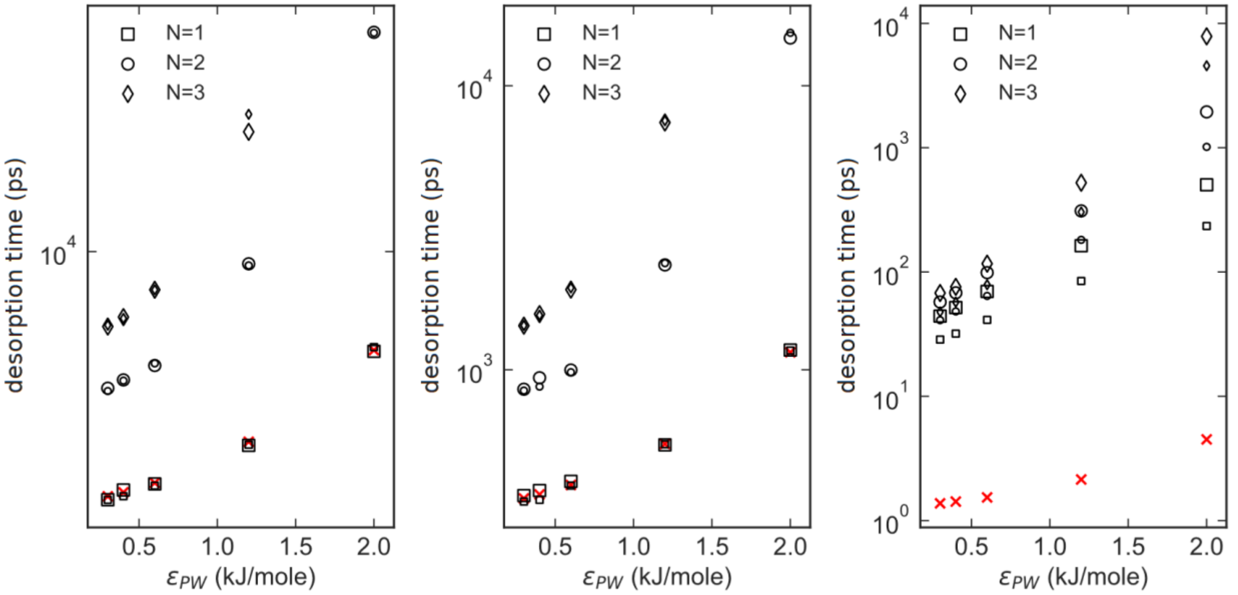

Figure S-2 Desorption times for monomers, dimers, and trimers at varying polymer-wall interaction energies and two bead masses. Similar plotting scheme to Figure S-1, but in this case large markers are for bead mass of 45 gm/mol, and small markers are for bead mass of 15 gm/mol, where in each sub-figure the friction coefficient ζ is held fixed at the values 12 x 10^{-10}, 3 x 10^{-10}, and 0.19 x 10^{-10} gm/s , from the left to the right sub-figures. For the simulations with ζ = 12 x 10^{-10} and 3 x 10^{-10}, decreasing the mass by a factor of 3 does not yield a discrepancy, whereas a discrepancy appears in the third sub-figure, where the friction coefficient is lowest, 0.19 x 10^{-10} gm/s.

Forward flux sampling details

We implemented forward flux sampling as outlined in the introduction with a control script in Python which managed LAMMPS sessions with the Plumed plugin. The control script would initiate a LAMMPS run whenever the number of active LAMMPS sessions dropped below the number of available cores, allowing the operating system to schedule the CPUs rather than binding each session to a core.

Analysis of transition paths

We used the networkx Python module to build a directed graph that recorded microstates and paths as nodes [27]. Each observed microstate at the first level was assigned a parent named *start*. The *attempt* paths of a microstate were assigned as children of the microstate. Paths which failed to reach the next level and returned to the starting basin were assigned *start* as a child. Paths which succeeded in reaching the subsequent (but not final) level were assigned the resulting entrance state as a child. Paths which reached the final level were assigned a child named *end*. Entrance state nodes in the graph carried information including the level and configurational data file. We used networkx to analyze the graph. For example, we could ask for all paths starting at *start* and ending at *end*, which would return all transition paths as a list of nodes {start, entrance state, path, entrance state, …, path, end}. We could then obtain the set of all microstates at level 0 with descendants at level $\boldsymbol{\lambda_n}$.

LAMMPS details

We used the *real* units scheme that operates with time in femtoseconds, length in Å, and energy in kcal/mole. We converted length units to nm and energies to k_BT for presentation of results. The process of equilibration followed by adsorption during the equilibration step in the simulations was controlled with a Python script utilizing MDAnalysis and calling LAMMPS with the Plumed plugin.
